# Functional and Cosmetic Outcomes of Pterional Craniotomy and Its Modifications: A Scoping Review

**DOI:** 10.7759/cureus.108862

**Published:** 2026-05-14

**Authors:** Taylor G Kreul, Haley Kenner, Katja Klosterman, Ritu Bhalerao, Akaash Surendra, Ibtesam Salahuddin, Peter Nakaji

**Affiliations:** 1 College of Medicine, The University of Arizona/Banner University Medical Center Phoenix, Phoenix, USA; 2 General Surgery, Mayo Clinic Rochester, Rochester, USA; 3 School of Life Sciences, Arizona State University, Tempe, USA; 4 College of Osteopathic Medicine, Rocky Vista University, Parker, USA; 5 Neurosurgery, Banner University Medical Center Phoenix, Phoenix, USA

**Keywords:** craniotomy techniques, mini-pterional craniotomy, pterional craniotomy, temporal hollowing, temporalis muscle atrophy

## Abstract

Pterional craniotomy is a widely used approach for anterior and middle fossa pathologies; however, violation of the temporalis muscle may result in cosmetic deformities and functional deficits, including temporal hollowing and frontalis weakness. Over the past decade, multiple surgical modifications have been proposed to minimize these complications. However, no consensus has been reached regarding the optimal technique. We conducted a Preferred Reporting Items for Systematic Reviews and Meta-Analyses (PRISMA)-guided review of recent literature evaluating temporalis-preserving or reconstructive approaches in pterional craniotomy. Across 18 studies involving 1,339 patients, both the mini-pterional and osteoplastic techniques were associated with superior cosmetic and functional outcomes compared with the traditional pterional approach, with notable advantages for specific aneurysm locations. These findings highlight the growing emphasis on quality of life after craniotomy and suggest that tailored technique selection may improve patient-centered outcomes.

## Introduction and background

The pterional craniotomy is a commonly utilized transcranial approach in neurosurgery. Named for its location at the pterion, this technique was formally described in 1975 by Mahmut Yasargil and is widely used for the treatment of anterior and middle cranial fossa pathologies, including ruptured and unruptured anterior circulation aneurysms as well as intracranial tumors [1]. The pterional approach provides broad basal exposure of the lateral sulcus and adjacent structures, typically involving drilling of the sphenoid wing, thinning of the orbital roof, and opening of the Sylvian fissure and basal cisterns [2].

Despite the widespread use of this approach, several complications commonly occur and remain a concern for both patients and providers. Many of these complications result from violation of the overlying temporalis muscle and associated neurovascular structures, including the facial and frontal nerves [3,4]. Damage to the temporalis muscle can result in facial asymmetry, discomfort with eyewear, temporomandibular joint (TMJ) dysfunction, pain with mastication, temporalis muscle hollowing, eyebrow droop, and facial nerve injury [3]. These complications are largely driven by disruption of the temporalis muscle’s vascular supply and innervation during dissection, resulting in denervation, ischemia, and subsequent muscle atrophy, which clinically manifests as temporal hollowing and facial asymmetry. The incidence of frontal nerve injury has been reported in up to 20% of pterional cases, while the incidence of TMJ dysfunction has been reported to be 37.5% [3,4]. Importantly, the incidence of temporal hollowing or atrophy after pterional craniotomy has been reported to be as high as 87% to 100% [5]. Atrophy of the temporalis muscle can result in cosmetic deformity, masticatory dysfunction, increased tension on other masticatory muscles, and reduced jaw stability. As the demand for minimally invasive techniques grows, numerous adaptations of the conventional pterional craniotomy have been developed in an effort to minimize these complications and improve both functional and cosmetic outcomes [3-5]. These techniques broadly aim to (1) reduce muscle dissection, (2) preserve neurovascular supply, or (3) reconstruct postoperative defects. First described in 1989, the osteoplastic pterional craniotomy involves preservation of the temporalis muscle on its bony attachment, with subsequent replacement of the osteoplastic flap after surgery, in an effort to restore the bony defect and reduce postoperative temporalis muscle atrophy and frontal nerve injury, thereby improving both functional and cosmetic outcomes. However, unique complications associated with osteoplastic craniotomy have been reported, including flap resorption and infection [6].

Another modified version of the traditional pterional craniotomy that has gained popularity is the mini pterional craniotomy. Formally described by Figueiredo et al., this technique utilizes a smaller bone flap and incision, along with reduced dissection of the temporalis muscle, compared with the standard pterional craniotomy [7]. These adaptations have been shown to decrease the incidence of temporalis muscle atrophy [8-11]. However, the mini pterional approach is not free from adverse outcomes, which still include frontal nerve injury and masticatory difficulties, albeit at lower rates than the conventional approach [9-11]. Potential drawbacks of the mini pterional craniotomy include a smaller surgical corridor; however, some studies report that the surgical exposure is comparable to that of the traditional pterional technique [9].

A third alternative is the frontolateral craniotomy, which involves a smaller incision compared with the pterional craniotomy in order to minimize muscle dissection and reduce postoperative temporalis muscle dysfunction. While generally safe, cerebrospinal fluid (CSF) leakage and bitemporal hemianopsia have been reported in rare instances [12]. Mini-temporal craniotomies offer another promising alternative for improved outcomes. This technique involves a smaller incision, which in turn leads to decreased muscle dissection. Reported complications associated with this method include transient postoperative hemiparesis [13].

As one of the most commonly used transcranial approaches in neurosurgery, continued refinement of the pterional craniotomy is essential to minimize known complications and improve patient satisfaction. Although many alternative techniques have been described, there remains a lack of consensus in the literature regarding which approach best optimizes clinical outcomes while minimizing functional and cosmetic deficits. Therefore, the aim of this review is to summarize the current state of the literature and present the reported complication rates associated with pterional craniotomy techniques that have gained popularity over the last decade.

This article was previously presented as a meeting abstract at the 2024 Congress of Neurological Surgeons Annual Scientific Meeting on September 30, 2024.

## Review

Methods

A scoping review was conducted in accordance with the Preferred Reporting Items for Systematic Reviews and Meta-Analyses (PRISMA) guidelines. A literature search was performed in PubMed, Embase, and the Cochrane Library in December 2023. Search terms included combinations of keywords and Boolean operators such as “pterional craniotomy”, “temporalis muscle”, “temporal hollowing”, and “craniotomy outcomes”.

Studies were included if they were published in English, involved patients undergoing pterional craniotomy, and reported functional and/or cosmetic outcomes associated with surgical technique. Studies were excluded if they involved pediatric or cadaveric populations, did not report relevant outcomes, focused on secondary reconstructive procedures, or were published before 2014.

**Figure 1:**
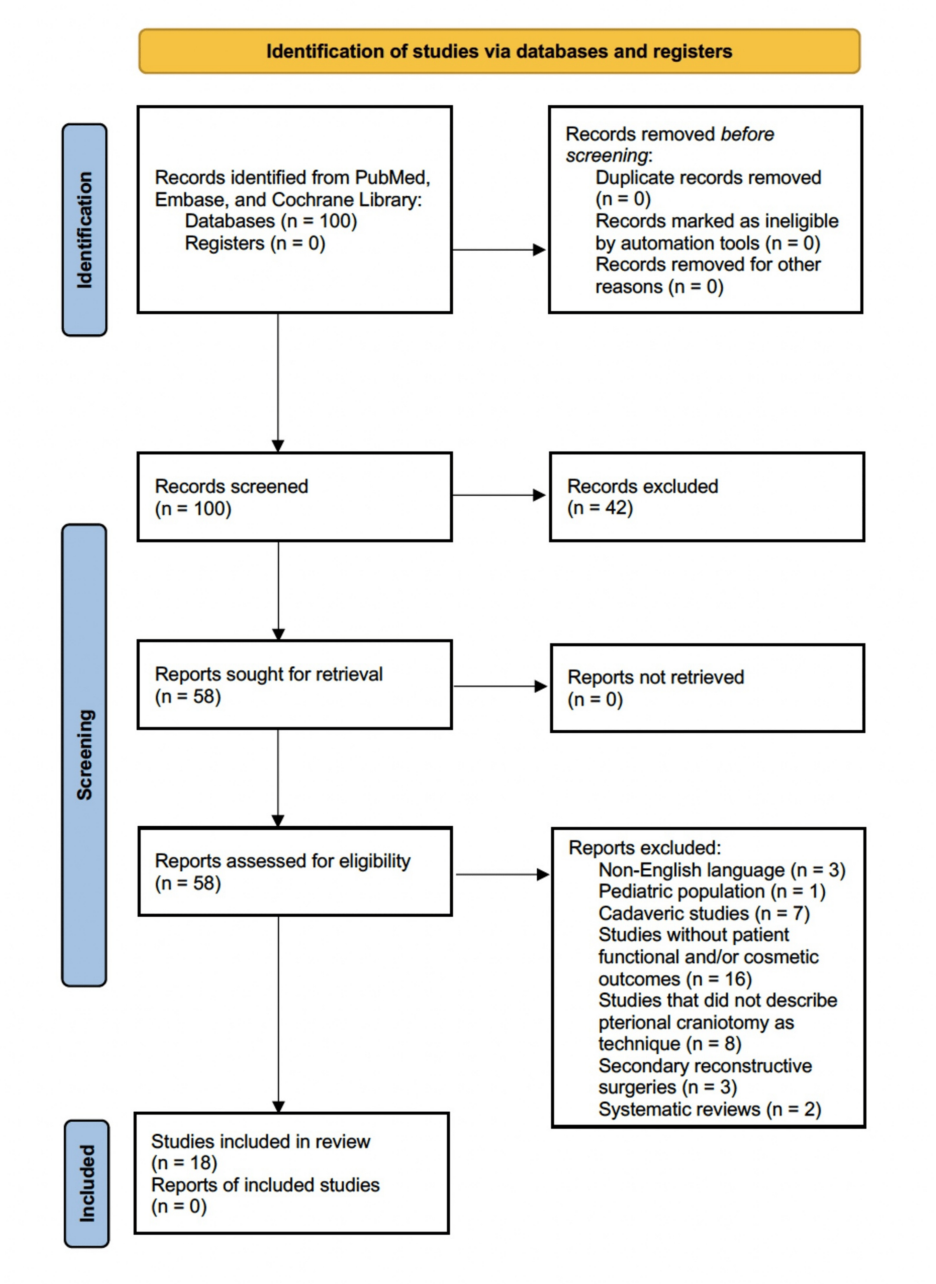
PRISMA diagram of the study selection process PRISMA: Preferred Reporting Items for Systematic Reviews and Meta-Analyses.

Titles and abstracts were screened by reviewers, and full-text articles were assessed for eligibility. Discrepancies were resolved through discussion and consensus. A PRISMA flow diagram summarizing study selection is provided in Figure 1. Data extracted from each study included study design, patient population, surgical technique, and reported outcomes.

Due to heterogeneity in study design, patient populations, and outcome reporting, the results were synthesized descriptively. As this study was designed as a scoping review, a formal risk-of-bias assessment and meta-analysis were not performed.

Results

Of the 100 articles identified and reviewed, 18 met the inclusion criteria and were selected for further review. Among these, 10 were retrospective reviews including 989 patients, 4 were prospective studies including 259 patients, 3 were technical notes including 86 patients, and 1 was a case series including 5 patients, for a total of 1,339 patients. A total of 18 studies were included and are summarized in Table 1.

**Table 1:** Characteristics of included studies evaluating pterional craniotomy variations This table summarizes the study design, publication year, and sample size of the 18 studies included in this systematic review. Studies encompass retrospective and prospective cohorts, technical notes, and case series evaluating conventional and modified pterional craniotomy techniques. Modified approaches include mini pterional, osteoplastic, frontolateral, and mini temporal craniotomies, as well as adjunctive reconstructive or preservation techniques aimed at improving functional and cosmetic outcomes.

Author (year)	Title	Journal	Design	No. of Subjects
Ajlan et al. (2024) [14]	A prospective comparison between soft tissue dissection techniques in pterional craniotomy: functional, radiological, and aesthetic outcomes	Operative Neurosurgery	Prospective review	78
Alkhalili et al. (2017) [10]	The mini pterional approach for ruptured and unruptured anterior circulation aneurysms: our initial experience	Asian Journal of Neurosurgery	Retrospective review	57
Caplan et al. (2014) [11]	The mini pterional craniotomy for anterior circulation aneurysms: initial experience with 72 patients	Neurosurgery	Prospective review	72
Kim et al. (2018) [15]	Temporal augmentation with calvarial onlay graft during pterional craniotomy for prevention of temporal hollowing.	Archives of Craniofacial Surgery	Retrospective review	100
Kim et al. (2016) [16]	Effectiveness of temporal augmentation using a calvarial onlay graft during pterional craniotomy	Archives of Plastic Surgery	Retrospective review	56
Kudulaiti et al. (2022) [3]	Mini temporal approach as an alternative to the classical pterional approach for resective temporal region surgeries	Chinese Neurosurgical Journal	Case series	5
Kweon et al. (2023) [17]	Acceptable durability of split inner table graft for the reconstruction of a bone defect in pterional craniotomies: a case series	Frontiers in Surgery	Retrospective review	9
Lee et al. (2022) [18]	Cosmetic outcome after electrocautery versus non-electrocautery dissection of the temporalis muscle for pterional craniotomy	Journal of Cerebrovascular and Endovascular Neurosurgery	Retrospective review	131
Park et al. (2015) [19]	A simple method for reconstruction of the temporalis muscle using contourable strut plate after pterional craniotomy: introduction of the surgical techniques and analysis of its efficacy	Journal of Cerebrovascular and Endovascular Neurosurgery	Retrospective review	106
Rai et al. (2022) [20]	Antegrade subperiosteal temporalis muscle elevation and posterior retraction technique avoiding muscle incision for pterional craniotomy: a technical note	Neurology India	Technical Note	50
Rai et al. (2022) [21]	Anchoring the temporalis muscle with an intact fascial layer along the superior temporal line in pterional craniotomy: a technical note	World Neurosurgery	Technical Note	30
Sanada et al. (2023) [22]	Rigid but nonmetallic cranioplasty after pterional craniotomy: technical note	Surgical Neurology International	Technical note	6
Seçer et al. (2022) [6]	Effects of modified osteoplastic pterional craniotomy on temporal muscle volume and frontal muscle nerve function	Journal of Neurological Surgery	Prospective review	51
Sriamornrattanakul et al. (2021) [4]	Suprafascial dissection for pterional craniotomy to preserve the frontotemporal branch of the facial nerve with less temporal hollowing	Surgical neurology international	Retrospective review	72
Sturiale et al. (2017) [9]	Mini pterional craniotomy for treatment of unruptured middle cerebral artery aneurysms. A single-center comparative analysis with standard pterional approach as regard to safety and efficacy of aneurysm clipping and the advantages of reconstruction	Acta Neurochirurgica	Retrospective review	68
Varol et al. (2023) [23]	Osteoplastic pterional craniotomy: success rate of surgery in patient aspect	Turkish Neurosurgery	Retrospective review	97
Welling et al. (2015) [8]	Prospective randomized study comparing clinical, functional, and aesthetic results of mini pterional and classic pterional craniotomies	Journal of Neurosurgery	Prospective review	58
Yang et al. (2014) [24]	The usefulness of the frontolateral approach as a minimally invasive corridor for clipping of anterior circulation aneurysm.	Journal of Cerebrovascular and Endovascular Neurosurgery	retrospective review	30

Detailed operative techniques, indications, and reported outcomes are presented in Table 2. 

**Table 2:** Surgical techniques, indications, and outcomes of pterional craniotomy variations This table summarizes the surgical techniques evaluated across included studies, associated pathologies, and reported functional (F) and cosmetic (C) outcomes. Techniques include conventional pterional craniotomy, mini pterional, osteoplastic, frontolateral, and mini temporal approaches, as well as adjunctive modifications such as onlay grafts, fixation systems, and dissection strategies. Reported outcomes focus on temporalis muscle atrophy, temporal hollowing, frontotemporal nerve function, temporomandibular joint dysfunction, and overall cosmetic satisfaction. Statistical significance is reported where available. AchoA: anterior choroidal artery, ACoA: anterior communicating artery, CSP: contourable strut plate, EC: electrocautery, FTL: frontolateral craniotomy, ICA: internal carotid artery, NEC: non-electrocautery, MC: myocutaneous flap, MCA: middle cerebral artery, MOPC: modified osteoplastic pterional craniotomy, MPT: mini pterional craniotomy, mRS: modified Rankin scale, PCoA: posterior communicating artery, PT: pterional craniotomy, TMJ: temporomandibular joint, VAS: visual analogue score.

Author (year)	Technique(s)	Pathologies treated	Outcomes	Key results
Ajlan et al. (2024) [14]	Pterional with 1 of 3 dissection techniques: myocutaneous flap, interfascial, or subfascial	Meningioma, high-grade glioma resection	C & F	No significant difference between groups in frontalis nerve function, TMJ dysfunction, temporalis muscle thickness, or cosmetic satisfaction. Temporal hollowing was more prominent in the myocutaneous flap group (p = 0.03)
Alkhalili et al. (2017) [10]	Mini pterional	Ruptured and unruptured MCA, ICA terminus, PCoA aneurysms	C & F	0/57 (0%) facial nerve damage or paresthesias around the incision line. 0/57 (0%) cranial disfigurement secondary to temporal hollowing or complications of incisional scar. 0/57 (0%) had pain or dysfunction of TMJ
Caplan et al. (2014) [11]	Mini pterional	Unruptured MCA, PCoA, paraophthalmic, AchoA, dorsal ICA aneurysms	C	Minimal to no temporalis muscle wasting was noted in 96% of patients
Kim et al. (2018) [15]	Pterional with or without temporal augmentation with split inner table onlay graft	Intracranial tumor excision of meningioma, glioma, schwannoma, craniopharyngioma, astrocytoma	C	Temporal thickness of the operated side showed statistically significant difference between augmentation and non-augmentation groups (p < 0.001). 93% of augmentation patients had no temporal hollowing. 95% of non-augmentation patients experienced temporal hollowing and 87% experienced discomfort. The augmentation group had a significantly lower VAS score than the non-augmentation group (p < 0.001). Two cases of temporal bulging reported the in augmentation group
Kim et al. (2016) [16]	Pterional with or without split inner table onlay graft	Tumor or hematoma removal	C	The mean VAS score was 1.4 ± 0.9 for the onlay graft group, while the mean score was 5.8 ± 2.2 for the pterional craniotomy group (p < 0.05). Preoperative soft tissue thickness was not significantly different between the operated and non-operated sides in the onlay group. The pterional craniotomy group showed a statistically significant difference between the thickness of the operated side and non-operated side (p = 0.006)
Kudulaiti et al. (2022) [3]	Mini temporal	Meningioma, glioblastoma, chordoma, astrocytoma resection	C & F	No postoperative complications related to extra-temporal cortical damage, atrophy of temporalis muscle, or injury to the facial nerve
Kweon et al. (2023) [17]	Conventional pterional and mini pterional with split inner table onlay graft	Pterional: ruptured AChoA, ACoA, MCA, PCoA. Mini pterional: unruptured AchoA, ACoA, MCA, PCoA	C	MPT was performed in three patients, PT was performed in six patients. Results were not reported separately. 2/9 (22%) patients exhibited a slight anterior temporal hollow due to temporalis atrophy. 7/9 (78%) no temporal hollowing. 0/9 (0%) exhibited focal depression at the frontobasal burr hole site. 0/9 (0%) exhibited infections of the bone graft. 3/9 (33%) patients demonstrated 21.2%-27.5% resorption of bone graft, but did not have temporal hollowing or cosmetic problems
Lee et al. (2022) [18]	Pterional (electrocautery vs non-electrocautery)	Not listed	C	Difference in incidence of depression at the mid-temporalis muscle was statistically significant in the EC group (25.6%) vs NEC group (2.6%) (p = 0.001). Patient surveys showed that cosmetic satisfaction was lower in the EC group vs NEC group (p = 0.002)
Park et al. (2015) [19]	Pterional with or without contourable strut plate	Cerebral aneurysms	C & F	Inferior displacement of temporalis muscle detected in 40/80 (50%) cases of MC-only group and 0/26 (0%) cases in the CSP group (p < 0.001). Depression along the inferior margin of the temporal line of the frontal bone was observed in 27/80 (34%) cases of MC-only group and 0/26 (0%) in the CSP group (p < 0.001). Muscular protrusion at the inferior portion of the temporal fossa observed in 20/80 (25%) cases in MC-only group and 0/26 (0%) CSP group (p = 0.001). 100% CSP had no cosmetic deformity; 63.8% of MC only had no cosmetic deformity (p <0.001).
Rai et al. (2022) [20]	Pterional with antegrade dissection using in-rolling vs out-rolling techniques	ACoA, MCA, PCoA, supraclinoid internal carotid aneurysms	C & F	0/50 (0%) facial nerve injury, temporalis-related complications, immediate periorbital edema; 50/50 (100%) excellent cosmesis at the six-month follow-up
Rai et al. (2022) [21]	Pterional and frontotemporal	ACoA, MCA, PCoA, temporal glioma, frontal glioma, insular glioma, meningioma, pituitary macroadenoma, craniopharyngioma, temporal epidermoid cyst, temporal metastatic lesion	C & F	The authors did not distinguish between pterional and frontotemporal craniotomy in the results. 0/30 (0%) immediate postoperative subgaleal collections, periorbital edema, temporalis muscle atrophy, or difficulty chewing or opening mouth at the six-month follow-up; 30/30 (100%) achieved excellent cosmetic and functional outcomes
Sanada et al. (2023) [22]	Pterional with CranioFix absorbable fixation system	MCA, internal carotid-anterior choroidal, paraclinoid aneurysms, and clinoidal meningioma	C	6/6 (100%) achieved bone fusion one year postoperatively. 0/6 (0%) cases exhibited observable deformity, asymmetry, sinking of the bone flap, or noticeable temporal region atrophy
Seçer et al. (2022) [6]	Pterional vs osteoplastic	Anterior circulation aneurysms	C & F	There was a statistically significant decrease in the temporal muscle volume in the PT group compared with the MOPC group (p < 0.001). Epidural hematoma developed in 1/30 (3%) of MOPC and in 2/21 (10%) cases of PT. No statistically significant relationship between MOPC and PT groups and the function of the frontal muscle nerve (p > 0.05)
Sriamornrattanakul et al. (2021) [4]	Pterional with suprafascial vs interfascial dissection	Cerebrovascular or tumor surgery	C & F	Frontalis paralysis evident in 11/54 (20%) suprafascial patients, but all recovered within six months postoperatively. Frontalis paralysis evident in 1/18 (6%) interfascial patients, which resolved within two months (p = 0.272). Obvious postoperative temporal hollowing evident in 18.2% of suprafascial group muscle cuff-off, 64.3% muscle cuff-on, overall 36.1% of suprafascial group, and 72.7% of interfascial group (significant p = 0.03)
Sturiale et al. (2017) [9]	Pterional vs mini pterional	Unruptured MCA aneurysms	C & F	7/37 (19%) PT patients had pain with mastication, compared with 1/31 (3%) in the MPT group (p = 0.06). 7/37 (19%) PT had functional limitations compared with 1/31 (3%) in the MPT group (p = 0.06). 29/31 (94%) MPT vs 30/37 (81%) PT with excellent cosmetic satisfaction (p = 0.16)
Varol et al. (2023) [23]	Pterional vs osteoplastic	Aneurysm	C & F	In conventional PT, mean muscle volume difference ratio of surgical vs non-surgical side was 31.88 ± 22.46, while in the osteoplastic PT, it was 8.15 ± 8.83 (p < 0.001). Conventional PT group had significantly lower cosmetic satisfaction levels than those who underwent osteoplastic (p < 0.001). 0/34 (0%) patients in the osteoplastic group developed postop hemorrhage, 3/63 (5%) patients in the conventional group developed postop hemorrhage
Welling et al. (2015) [8]	Pterional vs mini pterional	Ruptured and unruptured anterior circulation aneurysms	C & F	79% MPT vs 52% PT were satisfied with cosmesis (p = 0.07). Mean score for aesthetic rating was 27 in MPT vs 45.8 in PT group (p = 0.03). Photograph analyzers identified excellent and good results in 87% MPT vs 48% PT (p = 0.01). Measurements revealed atrophy in 12.7% MPT vs 22% PT group (p = 0.005). Functional outcomes, measured by mRS, were similar between PT and MPT groups at six months post-operatively (p = 0.99)
Yang et al. (2014) [24]	Pterional vs frontolateral	PT: unruptured MCA, ACA, ACoA. FTL: ACoA, PCoA, AChoA	C	Postoperative aesthetic outcome (by symmetry) was better in the FTL group (symmetric p = 0.002) than in the PT group (asymmetric p = 0.152) when measured by muscle thickness. 1/15 PC (7%) and 1/15 (7%) FTL developed epidural hematoma

Pterional Craniotomy

Description: The patient is positioned supine with the head rotated contralaterally, depending on lesion location. A curvilinear incision is made beginning anterior to the tragus and extending behind the hairline. The temporalis muscle is dissected to expose the cranial vault, and burr holes are placed to create a bone flap. The sphenoid wing is drilled to improve access to the anterior and middle skull base. The dura is opened, and the brain is gently retracted to access the target pathology. Following completion of the procedure, the bone flap is secured, and the temporalis muscle and skin are reapproximated [1,25].

Clinical indications: The pterional craniotomy is a workhorse in neurosurgery, allowing access to pathologies in the anterior and middle skull bases. Specifically, it provides access to lesions in the supratentorial basal cisterns and the superior portion of the infratentorial cisterns. For vascular lesions, this includes the internal carotid artery (ICA), middle cerebral artery (MCA), anterior communicating artery (ACoA), basilar tip, precommunicating (P1) segment of the posterior cerebral artery (PCA), and proximal segment of the superior cerebellar artery (SCA). This approach can also be utilized to address anterior and middle skull base lesions, orbital tumors, and intra-axial tumors in the frontal, temporal, and parietal lobes, including those within the uncus, insula, basal ganglia, lateral ventricle, and interpeduncular fossa [25]. The most common indications for pterional craniotomy include anterior circulation aneurysms and median and paramedian aneurysms involving the upper portion of the posterior circulation.

Strengths and weaknesses: The pterional craniotomy is widely used, mainly due to the extensive exposure it provides to a variety of intracranial structures. One key strength is its versatility, which stems from the ability to tailor the size of the bone flap and the extent of the craniotomy. Weaknesses include its invasiveness and the risk posed to important anatomical structures, such as the temporalis muscle, the frontotemporal branch of the facial nerve, the superficial temporal artery, and the auriculotemporal nerve. The associated functional and cosmetic outcomes resulting from injury to these structures are discussed below. 

Functional and cosmetic outcomes: There have been several adaptations to the traditional pterional craniotomy due to its suboptimal functional and cosmetic outcomes. One of the major complications is temporalis muscle atrophy, which has been reported to occur in 87% to 100% of cases [5]. This can cause significant cosmetic deformity as well as masticatory problems. Additionally, damage to the frontotemporal branch of the facial nerve can result in paralysis of the frontalis muscle. As previously mentioned, frontal nerve injury has been reported in up to 20% of pterional cases, while TMJ dysfunction has been reported in 37.5% [3,4].

In an effort to minimize damage to key structures such as the facial nerve, the pterional craniotomy can be performed using various dissection planes. Ajlan et al. reported no significant differences in frontalis nerve function or TMJ dysfunction when comparing the conventional pterional craniotomy performed with myocutaneous, interfascial, or subfascial dissection [14]. However, the authors reported that temporal hollowing was significantly more prominent when the pterional craniotomy was performed with myocutaneous dissection than with interfascial or subfascial dissection [14]. Sriamornrattanakul et al. reported no significant difference in frontalis paralysis when comparing the conventional pterional approach using suprafascial versus interfascial dissection [4]. However, they did report a significant difference in temporal hollowing between techniques, with 36.1% in the suprafascial group and 72.7% in the interfascial group experiencing temporal hollowing [4]. In 50 pterional craniotomy cases performed using an antegrade dissection technique, Rai et al. reported no cases of facial nerve injury or temporalis-related complications [20]. Rai et al. also reported in a separate study of 30 cases that temporalis muscle reattachment at the time of craniotomy was associated with good cosmetic and functional outcomes [21].

In addition to dissection plane selection, the use of electrocautery has been evaluated in pterional craniotomy. Lee et al. reported significant differences in both the incidence of temporalis muscle depression and cosmetic satisfaction when the pterional craniotomy was performed with versus without electrocautery, with the non-electrocautery group demonstrating better cosmetic outcomes and less depression [18].

Several studies have evaluated specific technical modifications to the pterional craniotomy. Park et al. evaluated the use of the contourable strut plate (CSP) in the conventional pterional approach [19]. The CSP used in this study was a titanium plate measuring 35 × 12.5 mm with a thickness of 0.4 mm and eight screw holes. Its utility lies in its malleability, allowing it to be contoured to the underlying bony surface [19]. This was one of the few studies included in this review to report a statistically significant difference in functional outcomes, concluding that CSP use reduces temporalis muscle inferior displacement and depression compared with the conventional pterional craniotomy. The authors also reported significantly fewer cosmetic deformities [19].

Another modification is the use of the CranioFix absorbable fixation system. This device assists in skull fixation at the conclusion of the pterional craniotomy. In contrast to titanium plates and other non-absorbable materials, such as poly-ether-ether-ketone (PEEK), CranioFix clamps provide an absorbable fixation option for cranioplasty. Sanada et al. reported a 100% rate of bone fusion and no cases of observable deformity, asymmetry, or temporal region atrophy in their analysis of six pterional craniotomy cases using the CranioFix absorbable fixation system [22].

Another modification described in the literature is the use of a split inner table onlay graft. Kim et al. found a significant reduction in temporal thickness loss in the non-augmented group compared with the augmented group [15]. Another study reported no significant difference in soft tissue thickness between surgical and non-surgical sides when a split inner table onlay graft was used [16]. In contrast, a significant difference in soft tissue thickness was observed in patients who did not receive the graft [16].

As summarized in Table 2, differences in functional outcomes were not statistically significant in the majority of studies comparing modified approaches with the conventional pterional craniotomy. In contrast, most studies reporting cosmetic outcomes demonstrated statistically significant improvements with modified techniques. These findings will be discussed in detail in the following sections for each craniotomy variation.

Mini Pterional Craniotomy

Description: A smaller arcuate incision is made along the anterior hairline, followed by subfascial or interfascial dissection to preserve the facial nerve. The temporalis muscle is elevated subperiosteally, and a limited craniotomy (typically ≤4 cm) is created [10,20]. The sphenoid ridge is drilled to enhance exposure, and the dura is opened to access the target pathology. After the procedure, the bone flap is replaced, and the temporalis muscle is reapproximated.

Clinical indications: The mini pterional craniotomy has been used as a reliable alternative to the traditional approach for the treatment of both ruptured and unruptured anterior circulation aneurysms, particularly for MCA, PCoA, and paraophthalmic aneurysms [7-10]. However, studies have suggested alternative approaches for distal MCA and distal anterior cerebral artery (ACA) aneurysms, reporting that the mini pterional approach may not provide adequate surgical access for these pathologies [10]. Specifically, Caplan et al. advise against the use of the mini pterional approach for ICA terminus or ACoA aneurysms due to the need for increased brain retraction [11]. Additionally, Alkhalili et al. do not recommend the mini pterional approach in cases of ruptured aneurysms associated with severe hemorrhage and massive edema requiring decompressive hemicraniectomy [10].

Strengths and weaknesses: The key strengths of the mini pterional craniotomy include its smaller size and shorter operative time, which may reduce disruption and ischemic exposure of key anatomic structures such as the temporalis muscle while still providing comparable surgical exposure to the standard pterional craniotomy [7,10,11]. Although smaller, the craniotomy allows access to similar anatomic targets [7]. Another advantage is that it follows natural anatomical planes, helping avoid injury to facial nerve branches and improving cosmetic outcomes [10]. Additionally, the mini pterional approach allows for improved surgical freedom and angular exposure due to the ability to tailor the craniotomy direction through sphenoid ridge drilling. With this customization, Figueiredo et al. found no statistically significant difference in surgical exposure between the pterional and mini pterional approaches [7]. Overall, this approach offers a balance between minimizing craniotomy size and maintaining adequate surgical exposure [7,9].

Functional and cosmetic outcomes: While not directly compared with the traditional pterional approach, Alkhalili et al. reported no cases of TMJ pain or dysfunction, facial nerve injury, or cranial deformity in their series of 57 mini pterional craniotomies [10]. In an analysis of 72 patients, Caplan et al. reported minimal to no temporalis muscle wasting in 96% of cases [11].

In direct comparisons between mini pterional and traditional pterional craniotomy, Sturiale et al. and Welling et al. both found no significant difference in functional outcomes, including pain and masticatory limitation [8,9]. Sturiale et al. reported excellent cosmetic satisfaction in 94% of cases but found no significant difference in cosmetic satisfaction compared with the pterional approach [9]. Welling et al. also found no significant difference in patient-reported cosmetic satisfaction between the two approaches [8]. However, when assessed by physician raters and photograph analysis, mini pterional patients scored significantly higher than pterional patients, indicating more favorable cosmetic outcomes and less atrophy [8].

Mini Temporal Craniotomy

Description: The patient is positioned supine with the head rotated contralaterally. A short curvilinear incision is made over the temporal region behind the hairline. The scalp and temporalis muscle are elevated together to preserve neurovascular supply, and a small craniotomy is created over the temporal squama [3,13]. The dura is opened to access the temporal lobe, with cerebrospinal fluid release used to facilitate exposure [3,13]. After the procedure, the bone flap and soft tissues are reapproximated.

Clinical indications: The mini temporal craniotomy is typically used for pathologies centered in the temporal lobe or middle cranial fossa. A primary indication for the technique is tumor resection of various temporal region lesions, such as temporal lobe gliomas, meningiomas, and lesions near the Sylvian fissure requiring open biopsy [3]. The mini temporal craniotomy has also been used in temporal lobe epilepsy surgery, such as anterior temporal lobectomy for drug-resistant epilepsy [13]. This approach is favored for lesions that do not require extensive exposure of adjacent regions, such as the frontal or parietal lobes, or major skull base work.

This approach is not appropriate when extensive exposure or multi-directional access is required. The mini temporal technique is also not ideal for tumors requiring aggressive skull base bone resection or lesions requiring extensive bony removal [3].

Strengths and weaknesses: Strengths of the mini temporal approach include minimal soft tissue trauma due to preservation of the facial nerve, and the use of a single-layer skin-galea-muscle flap, which may reduce the risk of frontalis muscle palsy and postoperative muscle atrophy [3]. The likelihood of postoperative cerebrospinal fluid (CSF) leakage and infection may also be reduced because the frontal sinus is not opened. Another advantage is the reduced risk of injury to uninvolved cortical regions due to more limited exposure compared with the classical approach. In addition, the smaller bone window may reduce operative time and decrease the risk of nerve injury compared with the pterional craniotomy [3].

Weaknesses include the limited surgical window, which may require frequent microscope adjustments when anatomical visualization is restricted [3]. This approach is also less suitable than the traditional pterional craniotomy when aneurysms require full Sylvian fissure dissection or when deep bony drilling is necessary [3].

Functional and cosmetic outcomes: A key motivation for selecting the mini temporal craniotomy is its potential for improved functional and cosmetic outcomes compared with more invasive approaches. Kudulaiti et al. reported no temporalis muscle atrophy, facial nerve injury, or postoperative complications related to extra-temporal cortical damage [3]. Patients in their study did not experience noticeable temporal hollowing postoperatively, supporting the cosmetic benefit of this minimally invasive technique [3]. However, these findings are based on a small cohort, and larger studies are needed to validate these advantages.

Frontolateral Craniotomy

Description: The patient is positioned supine with the head rotated away from the operative side. A small incision is made behind the hairline, and a skin-galea-muscle flap is elevated and retracted to expose the superior orbital rim [12,24]. A limited craniotomy is performed at the level of the orbital roof, and the dura is opened to access anterior skull base structures [12,24]. This approach allows targeted exposure while minimizing disruption of the temporalis muscle [24].

Clinical indications: Indications for the frontolateral approach include anteriorly based pathologies. Studies included in this review advocate its use for ACoA, PCoA, and AChoA aneurysms. However, in selected cases, larger tumors may also be accessible. Gerganov et al. reported a series of patients with giant craniopharyngiomas successfully resected using the frontolateral approach [12]. In these cases, tumor-related cranial expansion provided sufficient working space despite the limited craniotomy size. Similar applicability may exist for other large lesions causing skull expansion.

Strengths and weaknesses: The frontolateral approach offers several advantages. It allows for a shorter skin incision beginning just behind the hairline, resulting in a smaller craniotomy [24]. Despite its limited size, it provides adequate access to the anterior Circle of Willis and anterior basilar artery. Importantly, it spares the temporalis muscle, thereby reducing the risk of temporalis muscle atrophy [24]. Additionally, it has been associated with a lower incidence of postoperative epidural hematoma related to CSF leakage [24]. Collectively, these factors may contribute to shorter operative times and fewer postoperative complications compared with the pterional craniotomy.

Limitations of the frontolateral craniotomy are primarily related to its restricted exposure. It provides limited access to lesions requiring a more lateral trajectory, including certain PCoA, AChoA, MCA, and posterior basilar artery pathologies [24]. Aneurysms or lesions in these locations may be difficult to adequately visualize and manage using this approach.

Functional and cosmetic outcomes: Because the frontolateral approach does not disturb the temporalis muscle, it reduces the risk of temporalis muscle atrophy and associated facial asymmetry. This translates into improved cosmetic outcomes compared with conventional pterional craniotomy. In an analysis of 30 cases, Yang et al. found that postoperative aesthetic outcomes, as measured by symmetry, were significantly better in the frontolateral group than in the pterional group [24]. 

Osteoplastic Craniotomy

Description: The patient is positioned supine with the head rotated contralaterally. A curvilinear incision is made behind the hairline, and interfascial dissection is performed to preserve the facial nerve and vascular supply [23,26]. Burr holes are placed, and a bone flap is elevated with the temporalis muscle attached, maintaining its vascular supply [23,26,27]. The dura is opened to access the surgical target. Following the procedure, the osteoplastic flap is repositioned and secured.

Clinical indications: This flap is indicated as an alternative approach in cases where a standard pterional craniotomy would otherwise be used. Studies in this review specifically cited the osteoplastic pterional craniotomy for anterior circulation aneurysms [23,26,27].

Strengths and weaknesses: Schlitt and Quindlen first described the osteoplastic pterional craniotomy as advantageous due to preservation of blood flow from the temporalis muscle to the underlying bone, thereby reducing bone resorption [26]. This technique also results in significantly reduced temporalis muscle atrophy compared with the conventional pterional approach [23,26]. The osteoplastic pterional craniotomy maintains a similar operative viewing angle to a standard free bone flap technique because the basal cut across the sphenoid wing is made as low as possible [27]. The flap can also be extended anteriorly, posteriorly, or inferiorly to obtain additional subfrontal or subtemporal exposure. Additionally, this approach may be associated with risk to the frontalis branch of the facial nerve [6]. Reported complications include an increased risk of postoperative hemorrhage and infection [27].

Functional and cosmetic outcomes: The osteoplastic approach is associated with less temporalis muscle thinning compared with the conventional pterional craniotomy [6,23]. This helps preserve facial symmetry and generally improves cosmetic outcomes. Seçer et al. reported a statistically significant reduction in temporalis muscle volume loss in pterional craniotomy patients compared with those undergoing the modified osteoplastic pterional approach [6]. The authors also reported no significant difference in frontal branch nerve function between the two approaches [6]. Similarly, Varol et al. found a significantly smaller reduction in muscle volume with the osteoplastic approach [23]. Patients in the pterional group reported significantly lower cosmetic satisfaction than those who underwent the osteoplastic technique [23].

Discussion

Despite the advent of several modified techniques, this review demonstrates that the conventional pterional craniotomy, described in 15 of the 18 included studies, remains the most commonly utilized technique for pathologies requiring craniotomy in the temporal region. The second most commonly described variation was the mini pterional craniotomy. Studies evaluating the mini pterional approach have documented its efficacy in maintaining an adequate surgical window for the treatment of both ruptured and unruptured anterior circulation aneurysms [7-11]. Of note, Caplan et al. argued against the use of the mini pterional approach for ICA terminus or ACoA region aneurysms due to the need for increased brain retraction [11]. Other modified approaches, such as the osteoplastic, frontolateral, and mini temporal craniotomies, were primarily evaluated in the setting of unruptured anterior circulation aneurysms and selected sellar or anterior skull base tumors.

While many studies examined modified approaches, several also evaluated variations in temporalis muscle dissection techniques within the traditional pterional craniotomy. Ajlan et al. specifically evaluated myocutaneous, interfascial, and subfascial dissection [21]. They concluded that these techniques provide similar frontalis nerve outcomes but differ in other measures [21]. For example, myocutaneous dissection was associated with a significantly higher incidence of TMJ dysfunction and temporal hollowing compared with the other dissection planes. In another study, Rai et al. concluded that avoiding direct incision of the temporalis muscle through antegrade, subgaleal, subfascial, and subperiosteal dissection helps preserve muscle volume and neurovascular integrity [22]. Similarly, Sriamornrattanakul et al. provided evidence supporting suprafascial over interfascial dissection, as interfascial dissection was associated with a significantly higher incidence of temporal hollowing [4]. Collectively, these studies demonstrate that even within the traditional pterional approach, the choice of dissection plane can meaningfully influence functional and cosmetic outcomes.

Although functional complications occur less frequently than cosmetic issues, they remain clinically relevant and important to patients. As noted previously, differences in functional outcomes were not statistically significant in the majority of studies directly comparing conventional and modified approaches. However, several studies still reported trends toward improved functional outcomes, particularly TMJ function and facial nerve function, with modified techniques compared with the conventional pterional craniotomy. These findings suggest that modified approaches may be considered when feasible to help minimize complications.

Across the included studies, functional outcomes such as temporomandibular joint dysfunction and facial nerve injury were generally comparable between techniques. In contrast, cosmetic outcomes, particularly temporalis muscle atrophy and temporal hollowing, were more consistently improved with modified approaches compared with the traditional pterional craniotomy. Overall, these findings suggest that while functional preservation may be similar across techniques, cosmetic outcomes appear more sensitive to surgical approach and technical modification.

Limitations

This review has several limitations. First, only three databases (PubMed, Embase, and the Cochrane Library) were searched, which may have resulted in the omission of relevant studies indexed elsewhere. Second, the number of included studies was relatively small, and the included literature demonstrated significant heterogeneity in study design, patient populations, surgical techniques, and outcome reporting, limiting the ability to perform quantitative synthesis or direct comparisons. Third, no formal risk-of-bias assessment was conducted, as this study was designed as a scoping review. Additionally, variability in how functional and cosmetic outcomes were defined and measured across studies may affect the generalizability of the findings. Finally, most included studies were retrospective in nature, which may introduce selection bias and limit the strength of the conclusions.

## Conclusions

Patients and providers alike are seeking improved cosmetic and functional outcomes after pterional craniotomy, which may influence procedural choice. This review consolidates the available literature on variations of the pterional craniotomy and provides evidence that techniques such as the mini pterional, frontolateral, osteoplastic, and mini temporal craniotomies may reduce adverse cosmetic and functional outcomes associated with temporalis muscle manipulation during pterional craniotomy. These approaches may offer particular advantages for surgeries involving specific types of aneurysms and tumors. However, each approach has unique limitations that should be taken into consideration. Furthermore, the type of pathology to be addressed should be considered when selecting a specific technique. In summary, the traditional pterional craniotomy has evolved into multiple variations, allowing neurosurgeons today to align their surgical approach with specific pathological goals and optimal patient outcomes.
